# Correction: The unintended mitochondrial uncoupling effects of the FDA-approved anti-helminth drug nitazoxanide mitigates experimental parkinsonism in mice

**DOI:** 10.1016/j.jbc.2021.100864

**Published:** 2021-06-16

**Authors:** Niharika Amireddy, Srinivas N. Puttapaka, Ravali L. Vinnakota, Halley G. Ravuri, Swaroop Thonda, Shasi V. Kalivendi

VOLUME 292 (2017) PAGES 15731–15743

In the above article, an affiliation was missing from the affiliations list. The missing affiliation is the Academy of Scientific and Innovative Research (AcSIR), Ghaziabad-201 002, India. The corrected byline should read: “The unintended mitochondrial uncoupling effects of the FDA-approved anti-helminth drug nitazoxanide mitigates experimental parkinsonism in mice” Niharika Amireddy^‡¶1^, Srinivas N. Puttapaka^‡¶1^, Ravali L. Vinnakota^‡¶^, Halley G. Ravuri^§^, Swaroop Thonda^‡¶^, and Shasi V. Kalivendi^‡¶2^ From the Centre for ^‡^Chemical Biology and ^§^Pharmacology and Toxicology, Council of Scientific and Industrial Research (CSIR)–Indian Institute of Chemical Technology, Uppal Road, Tarnaka, Hyderabad – 500 007, Telangana State, India. ^¶^Academy of Scientific and Innovative Research (AcSIR), Ghaziabad-201 002, India.

